# Factors associated with diarrhoea and acute respiratory infection in children under-5 years old in Ghana: an analysis of a national cross-sectional survey

**DOI:** 10.1186/s12887-021-02546-x

**Published:** 2021-02-13

**Authors:** Paschal Awingura Apanga, Maxwell Tii Kumbeni

**Affiliations:** 1 University of Nevada, Reno, School of Community of Health Sciences, Reno, USA; 2 Ghana Health Service, Nabdam District Health Directorate, Nangodi, Ghana

**Keywords:** Diarrhoea, Acute respiratory infection, Children under-5 years, Ghana

## Abstract

**Background:**

Diarrhoea and acute respiratory infection (ARI) are major causes of morbidity and mortality in children under-5 years old in Ghana. The aim of the study was to assess factors associated with diarrhoea and ARI in children under-5 years old.

**Methods:**

We analysed nationally representative data from the 2017–2018 Ghana Multiple Indicator Cluster Survey (MICS) on 8879 children under-5 years old. Multivariable logistic regression was used to assess the factors associated with diarrhoea and ARI. We applied sample weights, stratification and clustering to account for the sampling design of the MICS.

**Results:**

The prevalence of diarrhoea was 17.0% (95% CI: 15.70, 18.24%). Children aged 6–11 months [Adjusted prevalence odds ratio (aPOR): 2.06, 95% CI: 1.45, 2.92], and 12–23 months (aPOR: 2.37, 95% CI: 1.67, 3.35), had higher prevalence of diarrhoea compared to children aged 0–5 months. Children whose mothers had a college or higher education (aPOR: 0.41, 95% CI: 0.22, 0.78), and a secondary education (aPOR: 0.66, 95% CI: 0.51, 0.86), had 59% and 34% lower odds of diarrhoea respectively, compared to children whose mothers had no formal education. Children from the richest households (aPOR: 0.58, 95% CI: 0.39, 0.86), had 42% lower odds of diarrhoea compared to children from the poorest households. Children resident in rural areas had 22% lower odds of diarrhoea compared to their peers in urban areas (aPOR: 0.78, 95% CI: 0.63, 0.98). The prevalence of ARI was 33.3% (95% CI: 31.72, 34.82%). Children aged 6–11 months (aPOR: 1.43, 95% CI: 1.06, 1.93), and 12–23 months (aPOR: 1.41, 95% CI: 1.10, 1.82), had higher prevalence of ARI compared to children aged 0–5 months.

**Conclusions:**

This study suggests that the prevalence of diarrhoea and ARI among children aged 6–11 and 12–23 months was higher compared to children aged 0–5 months. Children under-5 years old whose mothers had a secondary or higher education had a lower prevalence of diarrhoea compared to children whose mothers had no formal education.

## Introduction

Globally, diarrhoea and acute respiratory infections (ARIs) are major causes of mortality among children under-5 years old [1, 2]. Children in Sub-Saharan Africa are more than 15 times at risk of death before age 5 as compared to children in high income countries, with some of these deaths attributable to diarrhoeal disease and ARI [3]. Due to the survival threat posed by these health events, the Sustainable Development Goal 3 aims to reduce under-5 mortality to as low as 25 per 1000 live births by 2030 [4]. The World Health Organization (WHO) has also recommended for countries to provide all children with essential health services without undue financial hardship as part of measures to reduce under-5 morbidity and mortality [3].

In Ghana, diarrhoea and ARI are leading causes of under-5 mortality [5]. Diarrhoea and ARI are part of the top 10 causes of hospital admissions and mortality in children under-5 years old in Ghana [6]. Ghana’s under-5 mortality has declined from 82 per 1000 live births in 2011 to 56 per 1000 live births in 2018 [7, 8]. Whilst there seem to be a considerable decline in under-5 mortality, Kipp et al. observed that Ghana is among eight countries in Africa making little progress towards the reduction in under-5 mortality [9]. The high diarrhoea and ARI morbidities in children under-5 years of age are largely blamed for the slow pace in decline in under-5 mortality [10, 11]. Several policies and interventions have been implemented in Ghana including, the Free maternal health policy, Child health policy 2007–2015, Newborn care strategy 2014–2018, and Community-based health planning and services (CHPS) policy as part of measures to address the high under-5 morbidity and mortality in the country [12–15]. Therefore, assessing factors associated with diarrhoea and ARI would be relevant to Ghana where these health events are strongly associated with under-5 mortality [5, 6].

There are limited studies in Ghana on the prevalence and factors associated with diarrhoea and ARI in children under-5 years old [11, 16]. Many of these studies were not representative of children under-5 years in Ghana as they were restricted to selected districts and hospitals in the country [17–19]. Therefore we used data from the 2017–2018 Ghana Multiple Indicator Cluster Survey (MICS) [20], a national representative household survey, to assess factors associated with diarrhoea and ARI in children under-5 years old.

## Methods

### Study population

Our study population was children under-5 years old (0–59 months) in Ghana. We analysed data from the 2017–2018 MICS conducted in Ghana. The MICS is a national representative household survey conducted in many countries in the world with assistance from United Nations Children’s Fund (UNICEF), and provides robust data on women and children [21]. The MICS is a cross-sectional design, which employs a two-stage sampling technique which selects census enumeration areas from each sampling strata proportional to the number of households in an enumeration area. The second stage involves selection of households from each enumeration area to form survey clusters using systematic random sampling. Eligible mothers with children under-5 years old in selected households were interviewed [20, 22]. The response rate for eligible mothers with children under-5 years old was 99.7%. Details of MICS sampling procedures have been published [21].

### Primary outcomes

Our primary outcomes of interest were diarrhoea and acute respiratory infection (ARI). A child had diarrhoea if the mother or primary caretaker reported that the child had three or more loose or watery stools per day, or blood in stool two weeks prior to the survey. A child had ARI if the mother reported the child was ill with cough or difficulty in breathing [23], in the last two weeks. The term “mother” in our study refers to a biological mother or a female primary caretaker of the child under-5 years living in the same household.

### Primary variables of interest

We assessed whether child, maternal and household level factors were associated with diarrhoea and ARI in children under-5 years of age. These factors included: child’s age (0–5, 6–11, 12–23, 24–59 months); gender (boy, girl); child’s health insurance (yes, no); mother’s education (no formal education, primary, secondary, college or higher education); source of drinking water (improved, unimproved); sanitation (improved, unimproved); floor material (improved, unimproved); household wealth (poorest, poor, middle, rich, richest); place of residence (rural, urban); and presence of hand washing station in dwelling (yes, no).

We used the WHO/ UNICEF Joint Monitoring Programme for Water Supply, Sanitation and Hygiene (JMP) to categorize our source of drinking water [24]. An improved water source included source of drinking water from boreholes, piped water or tube wells, protected dug wells, protected springs, tanker-truck, rain water and packaged water, whilst an unimproved water source included unprotected springs, unprotected dug wells, and surface water collected directly from river, dam, lake, pond, stream, canal and irrigation channels. A household sanitation was considered unimproved if members of the household used pit latrines without a slab or platform, hanging latrines or bucket latrines, whilst improved sanitation included flush/pour flush toilet, pit latrine with slab, ventilated improved latrine and composting toilet according to JMP [25]. A household floor was considered improved if it was made up of cement, ceramic tiles, vinyl asphalt strips, parquet, polished wood, whilst floor materials such as earth, sand, dung, wood planks, palm, bamboo were considered unimproved [26]. Household wealth was categorized into wealth quintiles (poorest, poor, middle, rich, richest), and was determined using principal component analysis [27]. Our variable selection was guided based on previous studies [11, 16, 28], and data available in MICS.

### Data analysis

Data were analysed using descriptive statistics and complex survey multivariable logistic regression. Descriptive statistics were used to describe our study sample, and to assess the prevalence of our primary outcomes (diarrhoea and ARI) in our study sample.

Complex survey regression models (i.e. model for diarrhoea and a separate model for ARI) were used to assess the relationship between our primary variables of interest and primary outcomes. We conducted both univariate and multivariable logistic regression analyses for each of the primary outcomes. Univariate analysis was conducted for all primary variables in relation to their respective outcomes. Univariate analysis with a *P*-value of less than 0.2 was used to select variables into the multivariable regression models. Variables that had a *P*-value of greater than 0.2 at univariate analysis but were clinically relevant or might have some biological plausibility with the outcome were included in their respective multivariable regression model. We also tested for multicollinearity for each of the multivariable regression models using pairwise correlation matrix, variance inflation factor and tolerance, and eigensystem analysis of correlation matrix [29], to ensure that there was no multicollinearity issues. We assessed the fitness of each of our models using the global null hypothesis test. A *P*-value of less than 0.05 was considered statistically significant. Missing data was not a problem in our study as only the sanitation variable had missingness of 0.01% (i.e. only one child), which was dropped.

In all our descriptive statistics and regression analyses, we applied sample weights, stratification and clustering to account for the complex survey design, and to ensure the representativeness of the data. Data were analysed using SAS version 9.3 (SAS Institute, Cary, NC).

## Results

### Study sample

The study population and analytic sample was made up of 8879 children under-5 years old. The mean age of a child was 30 ± 7.3 months. One in 2 children were females (50.8%), and more than half of the number of children in our study had a health insurance (58.4%). Many of the children were living in the rural setting (56.9%) (Table 1). The overall prevalence of diarrhoea in our study was 17.0% (95% CI: 15.70, 18.24%), and the overall prevalence of ARI was 33.3% (95% CI: 31.72, 34.82%) [Results not shown].

**Table 1:** Characteristics of the study population (*n* = 8879)

Variable	N (%) or Mean (SD)
**Child age (months)**	30 (7.3)
**Child age (months)**	
0–5	830 (9.4)
6–11	871 (9.8)
12–23	1694 (19.1)
24–59	5483 (61.8)
**Gender**	
Boy	4370 (49.2)
Girl	4509 (50.8)
**Child’s health insurance status**	
Yes	5187 (58.4)
No	3692 (41.6)
**Mothers education**	
No formal education	2431 (27.4)
Primary	1792 (20.2)
Secondary	4213 (47.5)
College or higher education	443 (5.0)
**Source of drinking water**	
Unimproved	1386 (15.6)
Improved	7493 (84.4)
**Sanitation**	
Unimproved	3323 (37.4)
Improved	5556 (62.6)
**Floor material**	
Improved	8083 (91)
Unimproved	796 (9.0)
**Household Wealth**	
Poorest	1966 (22.1)
Poor	1834 (20.7)
Middle	1771 (19.9)
Rich	1678 (18.9)
Richest	1630 (18.4)
**Place of residence**	
Urban	3825 (43.1)
Rural	5054 (56.9)
**Presence of handwashing station in dwelling**	
No	8248 (92.9)
Yes	631 (7.1)

### Factors associated with diarrhoea and ARI

The multivariable regression analysis on diarrhoea showed that children aged 12–23 months and 6–11 months had 2.37 and 2.06 respectively, times the odds of diarrhoea compared to children aged 0–5 months. Children whose mothers had a secondary education, and a college or higher education had 34% and 59% lower odds of diarrhoea respectively, compared to children whose mothers had no formal education. Children from the richest households had 42% lower odds of diarrhoea compared to children from the poorest households [Adjusted prevalence odds ratio (aPOR): 0.58, 95% CI: 0.39, 0.86]. Children resident in rural settings had 22% lower odds of diarrhoea compared to those in urban settings (aPOR: 0.78, 95% CI: 0.63, 0.98) (Table 2).

**Table 2:** Factors associated with diarrhea in children under-5 years old (*n* = 8879)

Variable	Unadjusted OR (95% CI)	Adjusted OR (95% CI)
**Child age (months)**		
0–5	1	1
6–11	2.01 (1.44,2.82)	2.06 (1.45,2.92) *
12–23	2.31 (1.63,3.28)	2.37 (1.67,3.35) *
24–59	1.20 (0.85,1.69)	1.15 (0.82,1.63)
Gender **		
Boy	1	1
Girl	0.95 (0.80,1.13)	0.95 (0.80,1.13)
**Child’s health insurance status**		
Yes	1	1
No	1.14 (0.96,1.35)	1.02 (0.86,1.20)
**Mothers education**		
No formal education	1	1
Primary	0.89 (0.70,1.13)	0.91 (0.71,1.17)
Secondary	0.59 (0.47,0.75)	0.66 (0.51,0.86) *
College or higher education	0.27 (0.16,0.46)	0.41 (0.22,0.78) *
**Source of drinking water**		
Unimproved	1	1
Improved	0.64 (0.48,0.85)	0.78 (0.54,1.11)
**Sanitation**		
Unimproved	1	1
Improved	0.68 (0.58,0.81)	0.86 (0.71,1.05)
Floor material **		
Improved	1	1
Unimproved	1.14 (0.92,1.42)	0.84 (0.64,1.11)
**Household Wealth**		
Poorest	1	1
Poor	0.84 (0.69,1.02)	0.92 (0.74,1.14)
Middle	0.81 (0.61,1.09)	0.94 (0.64,1.37)
Rich	0.69 (0.53,0.90)	0.80 (0.58,1.12)
Richest	0.41 (0.31,0.53)	0.58 (0.39,0.86) *
**Place of residence**		
Urban	1	1
Rural	1.14 (0.94,1.39)	0.78 (0.63,0.98) *
**Presence of handwashing station in dwelling**		
No	1	1
Yes	0.48 (0.32,0.74)	0.74 (0.46,1.21)

* = Significant at *P*-value < 0.05; 1 = Reference category; ** = Variables with *P*-value > 0.2 but were included in the adjusted model as these variables are clinically relevant or might have some biological plausibility with the outcome

Our multivariable regression analysis on ARI revealed that children aged 6–11 months (aPOR: 1.43, 95% CI: 1.06, 1.93), and 12–23 months (aPOR: 1.41, 95% CI: 1.10, 1.82), had higher prevalence of ARI compared to children aged 0–5 months. All other variables were not associated with ARI (Table 3).

**Table 3:** Factors associated with acute respiratory infection in children under-5 years old (*n* = 8879)

Variable	Unadjusted OR (95% CI)	Adjusted OR (95% CI)
**Child age (months)**		
0–5	1	1
6–11	1.44 (1.07,1.93)	1.43 (1.06,1.93) *
12–23	1.44 (1.12,1.85)	1.41 (1.10,1.82) *
24–59	1.14 (0.92,1.43)	1.13 (0.90,1.41)
Gender **		
Boy	1	1
Girl	0.92 (0.8,1.06)	0.93 (0.80,1.06)
**Child’s health insurance status**		
Yes	1	1
No	0.96 (0.85,1.09)	0.94 (0.83,1.07)
**Mothers education**		
No formal education	1	1
Primary	1.25 (1.01,1.55)	1.22 (0.98,1.53)
Secondary	1.10 (0.96,1.27)	1.06 (0.90,1.24)
College or higher education	0.89 (0.63,1.26)	0.78 (0.53,1.15)
Source of drinking water **		
Unimproved	1	1
Improved	0.93 (0.79,1.09) **	0.84 (0.70,1.02)
**Sanitation**		
Unimproved	1	1
Improved	0.97 (0.85,1.11)	0.92 (0.79,1.07)
**Floor material**		
Improved	1	1
Unimproved	0.76 (0.59,0.98)	0.77 (0.58,1.02)
Household Wealth **		
Poorest	1	1
Poor	1.06 (0.86,1.30)	1.02 (0.81,1.27)
Middle	1.18 (0.94,1.48)	1.14 (0.88,1.47)
Rich	1.07 (0.87,1.33)	1.03 (0.78,1.34)
Richest	1.11 (0.89,1.38)	1.08 (0.81,1.43)
**Place of residence**		
Urban	1	1
Rural	0.91 (0.79,1.04)	0.89 (0.75,1.06)
Presence of handwashing station in dwelling **		
No	1	1
Yes	1.08 (0.82,1.44)	1.11 (0.80,1.56)

* = Significant at P-value < 0.05; 1 = Reference category; ** = Variables with *P*-value > 0.2 but were included in the adjusted model as these variables are clinically relevant or might have some biological plausibility with the outcome

## Discussion

Our study found that the prevalence of diarrhoea and ARI were 17.0% and 33.3% respectively. We also observed that children aged 6–11 and 12–23 months were associated with higher odds of diarrhoea and ARI. Children whose mothers had at least a secondary education, and children from the richest households were associated with lower odds of diarrhoea. Children resident in rural settings were associated with lower odds of diarrhoea.

The prevalence of diarrhoea in our study was lower than that reported by Amugsi et al. in their analysis of the 2008 Ghana Demographic and Health Survey (i.e. 20.9%) [11]. The prevalence of diarrhoea reported in our study indicates a slow decline in diarrhoea prevalence over the past 10 years in children under-5 years old, and may be part of the reasons why Ghana is making little progress towards the reduction of under-5 mortality [9]. Ghana may need to implement programmes focussed on diarrhoea reduction if a rapid decline is to be achieved.

With regards to ARI, the prevalence of ARI in our study was higher than that reported by Amugsi et al. in Ghana (22.4%), but lower than that reported in Nigeria (64.9%), Cameroon (54.7%), and India (41.6%) [11, 30–32]. The differences in prevalence of ARI might be attributed to variations in case definitions for ARI, child age, study population, study period and seasonality.

Our study also found that children aged 6–11 months and 12–23 months had higher odds of diarrhoea and ARI compared to their peers aged 0–5 months. This finding is consistent with many previous studies [11, 33–36]. Children within the ages of 0–5 months in Ghana are usually exclusively breastfed [8], and therefore our observed finding may be a reflection of the important role exclusive breast feeding plays in reducing diarrhoea and respiratory infection in children who are exclusively breastfed [37, 38]. The lower prevalence of diarrhoea among children aged 0–5 months may also be attributed to the innate immunity and less exposure to contaminated agents compared to children aged 6-23 months as children within this age group usually receive supplementary /complementary foods [38].

Our results also showed that children whose mothers had at least a secondary education had lower odds of diarrhoea compared to children whose mothers had no formal education. This is not surprising as education helps women to be well informed on how to access and apply information on child health. This finding in our study has been reported by other studies [16, 33]. We also found that children in the richest households had lower odds of diarrhoea compared to children in the poorest households. This finding is in conformity with previous studies in Ghana and Ethiopia [16, 39], but inconsistent with findings from Tanzania [40]. In our study, we also found that children resident in rural settings reported lower odds of diarrhoea compared to children in urban settings. This is consistent with the findings of Kumi-Kyereme and his colleague in Ghana. Our finding was also inconsistent with a recent systematic review in Ethiopia [41]. The finding in our study may be due to the urban health penalty, which posits that urban areas tend to concentrate poor people and expose residents to unhealthy environments, leading to a disproportionate burden of poor health [42]. This could possibly account for the higher prevalence of diarrhoea in children resident in urban areas compared to rural areas.

We also did not find an association between the presence of a handwashing station in the home and diarrhoea or ARI. The presence of a handwashing station may not necessarily reflect handwashing behaviour and may be the reason for our observed finding. Our findings were consistent with the findings of Kamm et al. in western Kenya [43]. There is mixed evidence on the role of a designated place for handwashing on diarrhoea or ARI prevalence in children. Whilst a randomized controlled trial in Bangladesh found no association between a handwashing station and an influenza-like illness or influenza [44], an observational study found that handwashing stations were associated with lower prevalence of respiratory infection in children [45]. A cluster-randomised controlled trial in Kenya reported that handwashing stations did not reduce childhood diarrhoea [46]. However, a systematic review reported that handwashing promotion may reduce diarrhoea in children [47].

Whilst we did not find an association between household wealth and ARI unlike in other studies [48], our findings was in agreement with the findings of Woldeamanuel and Gebreyesus in Ethiopia [49]. Similarly, we did not observe an association between a child’s mother’s level of education and ARI contrary to other studies [30, 48].

Our study had strengths and limitations. The national representativeness of MICS data allows for our findings to be generalizable to the entire country. However, our study had several limitations. Our primary outcomes were self-reported and therefore subject to recall bias. We expect recall bias to be similar between the exposed and unexposed primary variables. Self-reported primary outcomes in our study were not objectively verified (i.e. clinically confirmed) and could potentially overestimate or underestimate the true prevalence of ARI or diarrhoea in our study. Another limitation is that our findings cannot be interpreted causally due to the cross-sectional design of our data. One other limitation is that we could not control for seasonal variation of ARI and diarrhoea in our analysis [18, 50].

## Conclusion

Children aged 6–11 and 12–23 months had higher prevalence of diarrhoea/ARI compared to children aged 0–5 months. The prevalence of diarrhoea was also lower among children whose mothers had at least a secondary education compared to children whose mothers had no formal education.

## Data Availability

MICS data is publicly available at: https://mics.unicef.org/surveys

## Abbreviations

aPOR: Adjusted prevalence odds ratio
ARI: Acute respiratory infection
CHPS: Community-based health planning and services
JMP: World Health Organization / United Nations Children’s Fund Joint monitoring programme for water supply, sanitation and hygiene
MICS: Ghana Multiple Indicator Cluster Survey
UNICEF: United Nations Children’s Fund
WHO: World Health Organization
